# Dosimetry impact of distinct gating strategies in cine MR image‐guided breath‐hold pancreatic cancer radiotherapy

**DOI:** 10.1002/acm2.14078

**Published:** 2023-06-19

**Authors:** Yuyan Dong, Panpan Hu, Xiaoyang Li, Wei Liu, Bing Yan, Fei Yang, John Chetley Ford, Lorraine Portelance, Yidong Yang

**Affiliations:** ^1^ Department of Engineering and Applied Physics University of Science and Technology of China Hefei Anhui China; ^2^ Department of Radiation Oncology the First Affiliated Hospital of USTC, Division of Life Sciences and Medicine, University of Science and Technology of China Hefei Anhui China; ^3^ The Miller School of Medicine University of Miami Miami Florida USA

**Keywords:** breath hold, cine MRI, gating strategies, pancreatic cancer

## Abstract

**Purpose:**

To investigate the dosimetry effects of different gating strategies in cine magnetic resonance imaging (MRI)‐guided breath‐hold pancreatic cancer radiotherapy.

**Methods:**

Two cine MRI‐based gating strategies were investigated： a tumor contour‐based gating strategy at a gating threshold of 0–5% and a tumor displacement‐based gating strategy at a gating threshold of 3–5 mm. The cine MRI videos were obtained from 17 pancreatic cancer patients who received MRI‐guided radiation therapy. We calculated the tumor displacement in each cine MR frame that satisfied the gating threshold and obtained the proportion of frames with different displacements. We generated IMRT and VMAT plans using a 33 Gy prescription, and motion plans were generated by adding up all isocenter‐shift plans corresponding to different tumor displacements. The dose parameters of GTV, PTV, and organs at risk (OAR) were compared between the original and motion plans.

**Results:**

In both gating strategies, the difference was significant in PTV coverage but not in GTV coverage between the original and motion plans. OAR dose parameters deteriorate with increasing gating threshold. The beam duty cycle increased from 19.5±14.3% (median 18.0%) to 60.8±15.6% (61.1%) for gating thresholds from 0% to 5% in tumor contour‐based gating and from 51.7±11.5% (49.7%) to 67.3±12.4% (67.1%) for gating thresholds from 3 to 5 mm in tumor displacement‐based gating.

**Conclusion:**

In tumor contour‐based gating strategy, the dose delivery accuracy deteriorates while the dose delivery efficiency improves with increasing gating thresholds. To ensure treatment efficiency, the gating threshold might be no less than 3%. A threshold up to 5% may be acceptable in terms of the GTV coverage. The displacement‐based gating strategy may serve as a potential alternative to the tumor contour based gating strategy, in which the gating threshold of approximately 4 mm might be a good choice for reasonably balancing the dose delivery accuracy and efficiency.

## 1 INTRODUCTION

Radiation therapy aims to maximize dose sparing to healthy tissues while ensuring the prescription dose to the tumor target. In radiation dose delivery, respiration‐induced tumor motion could severely compromise dose coverage,^1^ resulting in reduced tumor control and/or increased normal tissue complications. Therefore, motion management during radiation therapy is necessary, particularly for tumors located in thoracic and abdominal regions.

To ensure target coverage, a margin accounting for the tumor motion can be added to the clinical target volume (CTV) to form the internal target volume (ITV), which encompasses the entire tumor motion path.^2^ However, the ITV approach results in a high dose delivered to the normal tissues surrounding the tumor target.^3^, ^4^ Respiratory gating is another way to manage tumor motion and can be performed using either external surrogates^5^, ^6^, ^7^, ^8^ or implanted fiducial markers.^9^, ^10^, ^11^ Commonly used devices based on external surrogates include, for example, the real‐time position management system by Varian and the surface optical guidance system by Vision RT.^12^ Surrogate‐based techniques depend on the reproducibility of the preestablished geometric relationship between the external surrogate motion and the internal tumor motion. However, the relationship may vary significantly interfraction and even intrafraction.^13^, ^14^ On the other hand, fiducial markers implanted directly in the tumor are a more accurate surrogate for tumor motion tracking, but they carry a risk of implantation complications.^15^, ^16^ Since the deep inspiration breath‐hold (DIBH) technique is a standard motion management strategy and is effective in decreasing the normal tissue complication probability,^17^, ^18^, ^19^ respiratory gating combined with DIBH has been widely used to better spare healthy tissues in radiation therapy for pancreatic cancer.^20^, ^21^, ^22^

Magnetic resonance imaging does not carry radiation dose and offers superior soft tissue contrast over cone beam CT.^23^, ^24^, ^25^ In addition, it can image any plane across the patient's body at a speed fast enough for tumor motion tracking. Therefore, MR‐guided radiation therapy (MRgRT) has emerged as a new radiation therapy strategy, with MRI providing static images for pretreatment patient positioning and cine images for in‐treatment tumor monitoring. However, MRgRT is a relatively new modality, and its clinical applications require rigorous evaluation and continuous optimization. Specifically, cine MRI guidance is still in its early phase, and the gating strategies based on dynamic MR images have yet to be optimized clinically. Therefore, the aim of this study was to investigate distinct gating methods based on either tumor contour or tumor displacement and their dosimetry effects at different gating thresholds in cine MRI‐guided breath‐hold pancreatic cancer radiation therapy.

## 2 METHODS AND MATERIALS

### 2.1 Cine MRI data

The cine MRI videos used for motion analysis and treatment planning guidance were from 17 pancreatic cancer patients who received stereotactic body radiation therapy (SBRT) on a 0.35 T MRgRT system (MRIdian, Viewray, Inc., Oakwood Village, Ohio, USA). Patients were treated with DIBH. Cine MRI in a single sagittal plane was acquired continuously to monitor the tumor movement using a balanced steady‐state free precession sequence (TrueFISP) throughout the entire treatment. The cine MRI scan protocol acquired single sagittal plane images 5 mm thick at 4 frames per second, with a field of view of 350×350 mm^2^ and spatial resolution of 3.5×3.5 mm^2^. The treatment and cine image acquisition were carried out simultaneously and completed in the same duration for each patient. The mean treatment time of 17 patients was 40.5±9.6 (39.9) min.

### 2.2 Tumor displacement calculation

To investigate the dosimetry effects due to respiratory motion, it is important to obtain the target displacement accurately. Ideally, the displacement calculated based on manual contours should be the ultimate standard. However, manual contouring is labor intensive and time consuming and has become an almost impossible approach. For instance, there were often more than 10 000 frames in the cine MR video acquired during a single treatment fraction. Therefore, we adopted a practical approach using the normalized cross correlation (NCC)‐based template matching algorithm to acquire the target displacement.^26^ The NCC‐based template matching algorithm is a practical solution in tumor tracking and has been validated in lung cancer patients.^27^ In clinical practice, to improve the contouring precision, the acquired cine MR images were interpolated to a finer spatial resolution of 0.37 mm x 0.37 mm from the original image spatial resolution of 3.5 mm x 3.5 mm.^21^ We investigated the uncertainty of the algorithm and independently validated it by benchmarking the centroid of the algorithm‐delineated target against that of manual contours delineated by two experienced clinicians. The validation was conducted on cine MR images for all 17 patients with 60 images per patient. The inter‐observer variability was further investigated to validate the NCC‐based template matching algorithm. The validation data included 350 cine MR images from seven patients (35 treatment fractions). Four experienced clinicians manually contoured the target and the contours were compared with the ground‐truth generated by the STAPLE algorithm which is a popular method to generate the “gold‐standard” contours.^28^, ^29^ The centroid distance between each individual contour and the generated ground‐truth contour was measured to assess the inter‐observer variability, which was then compared with the tracking error. The tumor displacement was calculated with the following steps:

Step 1: A rectangular template was drawn around the tumor in the cine MR image acquired at DIBH, in which the tumor was in the center of the gating boundary. Set the centroid of the gating boundary as the baseline position and the search area larger than the tumor movement area.

Step 2: The NCC coefficient was calculated according to equation (1).^30^ The location corresponding to the peak NCC coefficient was determined as the target position.

(1) $$c\;\left(u,v\right)=\frac{\sum_{x=u+1}^{u+N_{x}}\sum_{y=v+1}^{v+N_{y}}\left(f\left(x,y\right)-{\bar{f}}_{u,y}\right)\left(t\left(x-u,y-v\right)-\bar{t}\right)}{\sqrt{\sum_{x=u+1}^{u+N_{x}}\sum_{y=v+1}^{v+N_{y}}{\left(f\left(x,y\right)-{\bar{f}}_{u,v}\right)}^{2}\sum_{x=u+1}^{u+N_{x}}\sum_{y=v+1}^{v+N_{y}}{\left(t\left(x-u,y-v\right)-\bar{t}\right)}^{2}}}\;$$

where *t* denotes the template with size $N_{x}\times N_{y}$ selected in Step 1 and $\bar{t}$ denotes the mean value of *t*. (u, v) denote the template shift in the x and y directions, respectively. *f* denotes the matrix with the same size as the template in the target frame image, and $\bar{f}$ denotes the mean value of f(x,y).

Step 3: The displacement of the tracking target relative to its baseline position was calculated. Positive displacement was defined as the direction inferior to the baseline position in the superior‐inferior (SI) direction and anterior to the baseline position in the anterior‐posterior (AP) direction.

### 2.3 Treatment planning and motion plan generation

We generated both intensity‐modulated radiation therapy (IMRT) plans with 18 fields evenly distributed in 360°and volumetric modulated arc therapy (VMAT) plans with one single arc covering 360°by adopting an SBRT prescription of 33 Gy in five fractions for all 17 patients in a Pinnacle treatment planning system (TPS).^31^ To minimize the radiation dose to the duodenum, the duodenum was expanded by 3 mm to create a planning organ‐at‐risk volume (PRV). Then, the planning target volume (PTV) was generated by first adding a 4 mm isotropic margin to the gross tumor volume (GTV) and then subtracting the PRV. The patient characteristics are summarized in Table 1. The OAR dose constraints are summarized in Table 2.^32^

**TABLE 1:** Patient information.

Characteristics	Values (median and range)
Age	63.7 (32‐87)
Sex	10 males, 7 females
Prescription	33Gy/5 fractions
GTV (cc)	43.9 (4.1–307.0)
PTV (cc)	60.7 (10.0–300.2)

**TABLE 2:** SBRT dose constraints for OARs.

OARs	Constraint
Duodenum	$D_{max}<32Gy$
	$V_{12.5Gy}<10\mathrm{cc}$
	$V_{18Gy}<5cc$
Stomach	$D_{max}<32Gy$
	$V_{18Gy}<10cc$
Small bowel	$D_{max}<30Gy$
	$V_{25Gy}<5cc$
Liver	$V_{<21Gy}>700cc$
Kidney L	$D_{mean}<10Gy$
Kidney R	$D_{mean}<10Gy$
Spinal cord	$D_{max}<30Gy$
	$V_{23Gy}<0.035cc$
	$V_{14.5Gy}<1.2cc$

**FIGURE 1:**
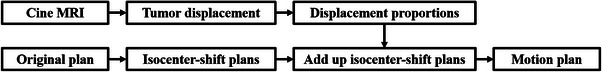
The workflow for motion plan generation.

To evaluate the motion impact on the dose distribution, we adopted an isocenter‐shift strategy to generate motion plans. First, we sorted out the MR frames that satisfied the predefined gating threshold and calculated the target displacement in the SI direction and AP direction. Second, we calculated the proportions of cine MR frames corresponding to different displacements over all the beam‐on frames for every treatment fraction. The proportions were also the time proportions of different displacements during the entire beam‐on period. Then, we averaged the time proportions across the five fractions. Finally, we shifted the isocenter by corresponding displacements and recalculated the radiation dose to generate isocenter‐shift plans and added up all isocenter‐shift plans according to their time proportions to create motion plans. The motion plan generation workflow is shown in Figure 1.

### 2.4 Motion management strategies

#### 2.4.1 Tumor contour‐based gating strategy

**FIGURE 2:**
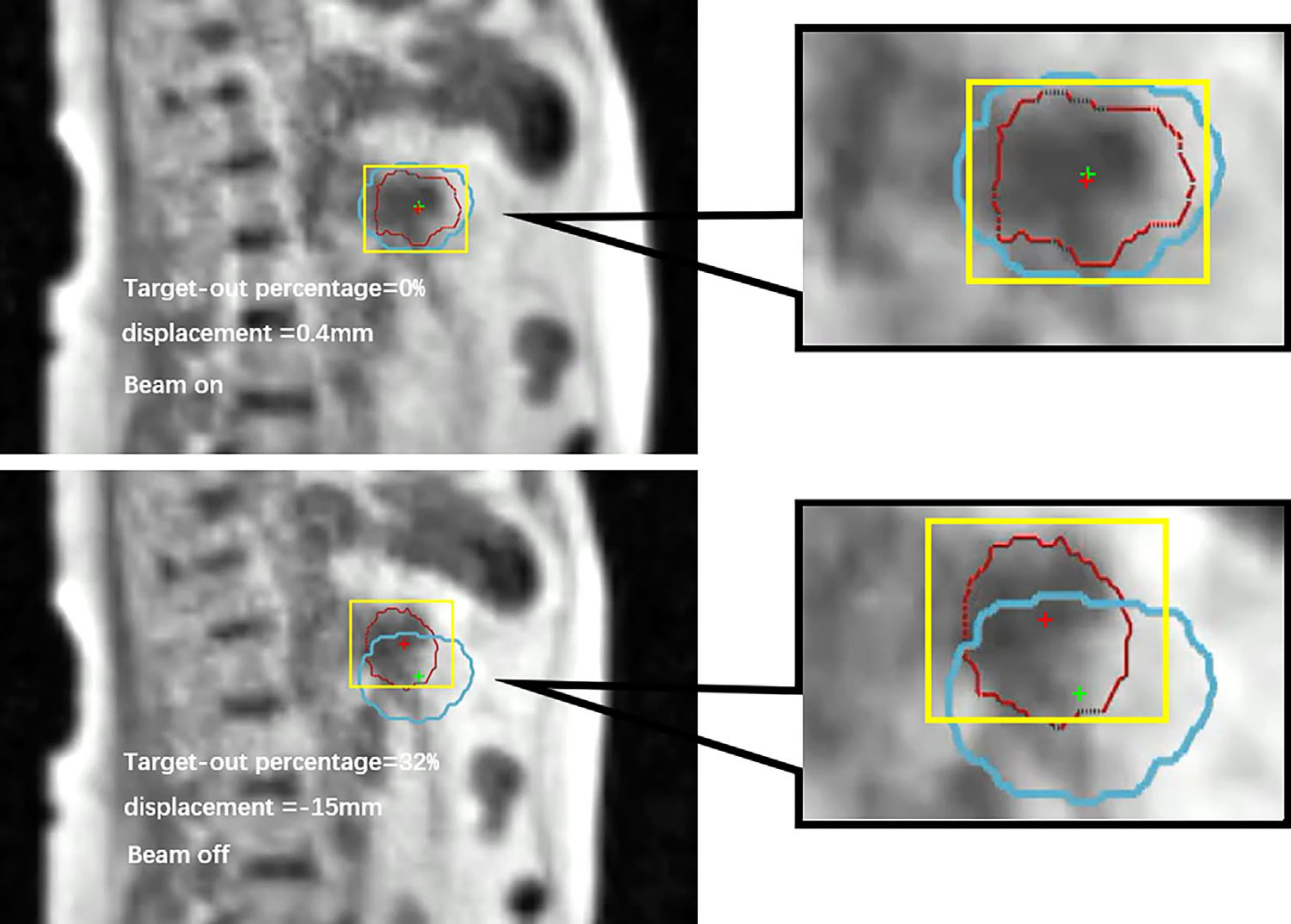
Two cine MRI‐based tumor gating strategies. In the tumor contour‐based gating strategy, the radiation beam is on when GTV (red contour) is inside the gating boundary (blue contour). Radiation is turned off when the target‐out percentage exceeds a predefined threshold, i.e., 5%. In the tumor displacement‐based gating strategy, the centroid of the tracking area (yellow box) is defined as the tracking position (red cross), and radiation is delivered only when the tracking position displacement is less than a predefined gating threshold, i.e., 4 mm. The green cross indicates the baseline position. The right panels show the enlarged area surrounding the tracking area.

The MRIdian system adopts a tumor contour‐based gating strategy. A setup 3D MR image was acquired prior to the dose delivery, from which a sagittal slice was localized for the subsequent 2D cine MR imaging. In the cine images, the tracking target was set to the GTV, and a margin of 3–5 mm around the GTV was used as the gating boundary. A target‐out percentage was calculated as the percentage of the GTV exceeding the tracking boundary. Radiation was delivered when the GTV was located within the gating boundary or the target‐out percentage was lower than a predetermined gating threshold. The target was auto‐contoured in real time by deforming the contour in the reference frame to the current motion frame.^20^, ^23^ An example of the tumor contour‐based gating strategy is demonstrated in Figure 2.

In the tumor contour‐based gating strategy, a target‐out percentage of 5% is usually used as the gating threshold in our clinic to ensure target coverage and avoid a treatment period that is too long. The gating threshold represents the allowed maximal target‐out percentage, and some studies found that it might be relaxed to a value up to 10%.^20^, ^21^ A greater threshold would increase the beam duty cycle and avoid a treatment period that is too long. However, we implemented a more conservative strategy and used the 5% threshold in our clinic to first ensure treatment accuracy. Therefore, a gating threshold of 0–5% was used in the evaluation of the tumor contour‐based gating strategy in the current study.

A worst‐case scenario was also examined by assuming that the target‐out percentage was always around the gating threshold. To create the worst‐case scenario plan, we shifted the isocenter of the original plan by the 90^th^ quantile of the displacements derived from those frames corresponding to the exact 5% target‐out percentage. The reason for using the 90^th^ quantile was to avoid the random use of the single maximum displacement, which might be too large.

#### 2.4.2 Tumor displacement‐based gating strategy

In the tumor contour‐based gating strategy, the actual dose delivered to the PTV and OARs depends on the contouring accuracy. However, how the contouring accuracy affects the delivered dose is still unclear. Therefore, the tumor displacement was acquired by the NCC‐based template matching algorithm to generate motion plans, which would independently validate the tumor contour‐based gating strategy. Intuitively, we also proposed a displacement‐based gating strategy as an alternative to the tumor contour‐based gating strategy. In tumor displacement‐based gating, a beam‐off event would be triggered immediately once the target displacement was greater than a predefined gating threshold. An example of the tumor displacement‐based gating strategy is demonstrated in Figure 2. Considering that the typical recommendation for the gating boundary was no greater than 5 mm for breath‐hold treatment,^2^, ^33^ gating thresholds of 3–5 mm were evaluated in this study.

### 2.5 Statistical analysis

Before a statistical hypothesis test, the normality of the data was required be tested first. In this study, the normality test was conducted based on the Kolmogorov–Smirnov test.^34^ After the Kolmogorov–Smirnov test, we found that most statistical distributions were not normal, so we performed the paired Wilcoxon signed rank test,^35^ which is often used to compare the matched samples with non‐normal distribution. The p value was corrected by Bonferroni correction by multiplying the number of statistical tests. The statistical analysis was based on p values after the correction. In addition, we compared the motion plans at different gating thresholds using the nonparametric Friedman test. The significance level was set at *P* < 0.05. The data is presented as the mean ± standard deviation (median). The coverage of GTV, PTV, and dose to OARs such as the duodenum, stomach, small bowel, liver, kidneys, and spinal cord were all evaluated to assess the impact of the different gating thresholds on the dose delivered.

## 3 RESULTS

### 3.1 The uncertainty of the tracking algorithm

Based on the manual contours delineated in cine MR images, the mean motion amplitudes for all 17 patients were from 11.22–22.59 mm in SI direction and 3.57–12.23 mm in AP direction. The tracking error for the NCC‐based template matching algorithm was 0.62 ± 0.25 (0.55) mm and 0.57 ± 0.28 (0.49) mm in SI and AP direction, respectively, and was much smaller than the gating threshold used in the displacement‐based gating strategy. According to the inter‐observer variability calculation, the centroid distance in SI and AP direction was 0.76 ± 0.65 (0.59) mm and 0.70 ± 0.63 (0.58) mm, respectively, and was comparable to the tracking error.

### 3.2 Dose comparison for IMRT plans

**FIGURE 3:**
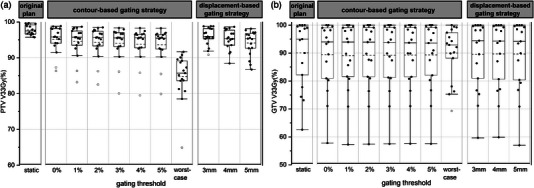
V33 Gy of the PTV (a) and GTV (b) of the IMRT plans at different gating thresholds in the tumor contour‐based and tumor displacement‐based gating strategies. The top and bottom of the box indicate the 25th and 75th percentile and the circle indicates the outlier. The mean and medium value are indicated by dotted line and solid line, respectively.

The PTV V33Gy and GTV V33Gy of the original and motion IMRT plans for 17 patients when applying different gating thresholds are shown in Figure 3. The GTV V33Gy was smaller than the PTV V33Gy because very often the GTV was close to the duodenum and the PTV was cropped by the duodenum PRV, which means some of the GTV was also cropped out and not included in the PTV coverage optimization. Table 3 presents the statistical comparisons between the original plan and motion plans. The results demonstrated a statistically significant decrease in PTV coverage and spinal cord Dmax for motion plans when compared to the original plan. A significant decrease was also found for the small bowel Dmax but only at the 0% gating threshold. No significant difference was found for other parameters. For the worst‐case scenario, significant differences were found for PTV V33Gy, duodenum Dmax, duodenum V12.5Gy, duodenum V18Gy, stomach V18Gy, and small bowel Dmax.

**TABLE 3:** Dosimetry comparisons for IMRT plans.

			Tumor contour‐based gating strategy							Tumor displacement‐based gating strategy		
		Original plan	0%	1%	2%	3%	4%	5%	worst	3 mm	4 mm	5 mm
		Mean±SD	Mean±SD	Mean±SD	Mean±SD	Mean±SD	Mean±SD	Mean±SD	Mean±SD	Mean±SD	Mean±SD	Mean±SD
	Parameter	(Median)	(Median)	(Median)	(Median)	(Median)	(Median)	(Median)	(Median)	(Median)	(Median)	(Median)
GTV	V33Gy (%)	90.1±11.9	89.5±12.3	89.1±12.3	89.1±12.3	89.2±12.2	89.3±12.2	89.3±12.2	90.0±9.5	89.6±12.5	89.7±12.4	89.7±12.7
		(95.0)	(94.0)	(94.0)	(93.9)	(93.8)	(93.7)	(93.6)	(92.9)	(94.5)	(94.3)	(94.2)
	*P* value_corr_		1.000	1.000	1.000	1.000	1.000	1.000	1.000	0.494	1.000	1.000
PTV	V33Gy (%)	97.8±1.4	95.1±3.8	94.6±4.3	94.3±4.4	94.0±4.7	93.8±4.8	93.7±4.7	84.8±6.4	96.0±2.5	95.2±2.9	94.1±3.5
		(97.5)	(95.8)	(95.7)	(95.6)	(95.6)	(95.6)	(95.5)	(85.7)	(95.9)	(95.4)	(95.4)
	*P* value_corr_		**<0.001**	**<0.001**	**<0.001**	**<0.001**	**<0.001**	**<0.001**	**<0.001**	**<0.001**	**<0.001**	**<0.001**
Duodenum	Dmax (cGy)	3105±86	3092±126	3095±131	3098±132	3099±132	3100±133	3100±134	3507±158	3101±111	3105±116	3102±134
		(3119)	(3117)	(3112)	(3111)	(3116)	(3122)	(3126)	(3543)	(3146)	(3160)	(3164)
	*P* value_corr_		1.000	1.000	1.000	1.000	1.000	1.000	**<0.001**	1.000	1.000	1.000
	V12.5Gy (cc)	9.4±1.2	9.7±1.6	9.9±1.9	10.0±1.9	10.0±1.9	10.0±1.9	10.0±1.9	13.6±4.7	9.7±1.4	9.8±1.6	9.9±1.7
		(9.3)	(10.0)	(9.9)	(10.0)	(10.0)	(10.0)	(10.0)	(12.0)	(10.1)	(10.2)	(10.3)
	*P* value_corr_		1.000	0.984	0.635	0.569	0.448	0.407	**0.013**	0.258	0.273	0.395
	V18Gy (cc)	3.5±0.6	3.6±0.9	3.7±0.9	3.7±1.0	3.8±1.0	3.7±1.0	3.8±1.0	7.1±3.5	3.6±0.8	3.7±0.8	3.7±0.9
		(3.3)	(3.4)	(3.6)	(3.6)	(3.6)	(3.6)	(3.6)	(7.7)	(3.5)	(3.5)	(3.5)
	*P* value_corr_		1.000	1.000	1.000	1.000	0.958	0.961	**0.003**	0.490	0.360	0.817
Stomach	Dmax (cGy)	2668±603	2651±581	2657±586	2659±591	2658±593	2655±594	2653±596	2783±760	2665±591	2661±595	2658±598
		(2749)	(2742)	(2744)	(2739)	(2738)	(2740)	(2742)	(2983)	(2744)	(2740)	(2743)
	*P* value_corr_		0.984	1.000	1.000	1.000	1.000	1.000	1.000	1.000	1.000	1.000
	V18Gy (cc)	5.0±3.7	4.9±3.7	4.9±3.8	4.9±3.8	4.9±3.8	4.9±3.8	4.9±3.8	6.8±6.6	4.0±3.8	5.0±3.8	5.0±3.8
		(5.3)	(5.0)	(5.1)	(5.1)	(5.1)	(5.1)	(5.1)	(4.8)	(5.2)	(5.3)	(5.2)
	*P* value_corr_		0.830	1.000	1.000	1.000	1.000	1.000	**<0.001**	1.000	1.000	1.000
Liver	V21Gy (cc)	1548±502	1548±502	1548±502	1548±502	1548±502	1548±502	1548±502	1550±501	1548±502	1548±502	1549±502
		(1457)	(1457)	(1457)	(1457)	(1458)	(1458)	(1458)	(1458)	(1458)	(1458)	(1458)
	*P* value_corr_		1.000	1.000	1.000	0.625	0.625	0.625	0.938	1.000	1.000	1.000
Small bowel	Dmax (cGy)	2458±703	2430±687	2429±689	2430±692	2431±694	2433±696	2436±698	2636±777	2432±692	2433±695	2436±698
		(2875)	(2883)	(2880)	(2885)	(2888)	(2892)	(2896)	(2928)	(2890)	(2894)	(2900)
	*P* value_corr_		**0.041**	0.089	0.155	0.359	0.657	1.000	**0.021**	0.116	0.302	0.962
	V25Gy (cc)	0.5±0.6	0.5±0.5	0.5±0.5	0.5±0.5	0.5±0.5	0.5±0.5	0.5±0.5	1.3±2.0	0.5±0.5	0.5±0.5	0.5±0.6
		(0.2)	(0.1)	(0.1)	(0.1)	(0.1)	(0.1)	(0.1)	(0.4)	(0.1)	(0.1)	(0.1)
	*P* value_corr_		1.000	1.000	1.000	1.000	1.000	1.000	1.000	1.000	1.000	1.000
Left kidney	Dmean (cGy)	412±19	409±187	410±187	410±187	410±187	410±187	410±187	419±192	409±187	410±187	410±187
		(398)	(396)	(397)	(397)	(397)	(397)	(397)	(410)	(398)	(398)	(399)
	*P* value_corr_		1.000	1.000	1.000	1.000	1.000	1.000	1.000	1.000	1.000	1.000
Right kidney	Dmean (cGy)	427±176	425±174	425±175	425±176	425±176	425±176	426±175	453±175	425±175	426±175	426±175
		(389)	(385)	(386)	(386)	(387)	(387)	(387)	(385)	(389)	(391)	(388)
	*P* value_corr_		1.000	1.000	1.000	1.000	1.000	1.000	0.507	1.000	1.000	1.000
Spinal Cord	Dmax (cGy)	984±367	978±365	977±364	977±364	976±363	976±363	975±363	977±367	976±364	975±363	974±362
		(918)	(917)	(917)	(916)	(916)	(915)	(915)	(907)	(917)	(916)	(916)
	*P* value_corr_		**0.007**	**0.008**	**0.007**	**0.007**	**0.006**	**0.006**	1.000	**<0.001**	**0.001**	**0.003**

*Note*: *P* value_corr_ is the *P* value after Bonferroni correction. The *P* values of <0.05 were presented in bold.

Table 4 summarizes the *P* values of PTV V33Gy comparisons between each two gating thresholds in the tumor contour‐based gating strategy. As shown, when the gating threshold was greater than 2%, the PTV V33Gy was significantly lower than that at the 0% gating threshold. No significant difference was found for PTV coverage between any two gating thresholds within 2%–5%. The worst‐case scenario plan was significantly different from all the motion plans except for the 5% gating threshold. In the tumor displacement‐based gating strategy, the 4 mm gating threshold resulted in a PTV coverage significantly better than the 5 mm threshold (*P* = 0.049) but worse than the 3 mm threshold (*P* = 0.011).

**TABLE 4:** PTV V33Gy comparisons between motion IMRT plans at different gating thresholds in the tumor contour‐based gating strategy.

Gating threshold	1%	2%	3%	4%	5%	Worst‐case
0%	1.000	0.054	**0.002**	**<0.001**	**<0.001**	**<0.001**
1%		1.000	0.090	**0.010**	**0.001**	**<0.001**
2%			1.000	1.000	0.674	**<0.001**
3%				1.000	1.000	**0.004**
4%					1.000	**0.041**
5%						0.233

*Note*: The *P* value of <0.05 were presented in bold.

**FIGURE 4:**
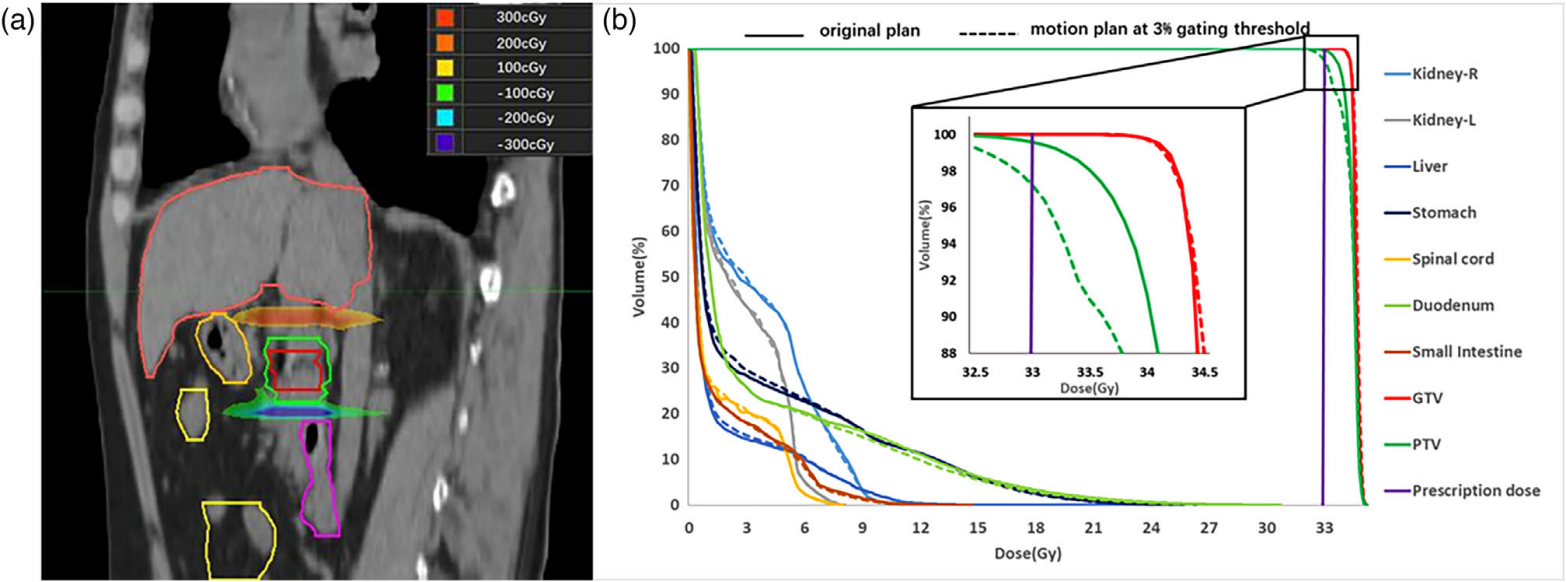
The change in dose distribution for an IMRT motion plan at the 3% gating threshold. (a) The dose difference between the motion plan and the original plan; (b) the DVHs of the original plan (solid line) and the motion plan (dashed line).

As an example, Figure 4a shows the dose difference between the motion plan at a 3% gating threshold and the original plan. As observed, the major difference occurred in the superior and inferior boundaries of the GTV and PTV, resulting in underdose at the inferior boundary and overdose at the superior boundary in the motion plan. Figure 4b shows the dose volume histogram (DVH) of the original plan and the motion plan. The difference was obvious for the PTV but not for the GTV.

### 3.3 Dose comparison for VMAT plans

**FIGURE 5:**
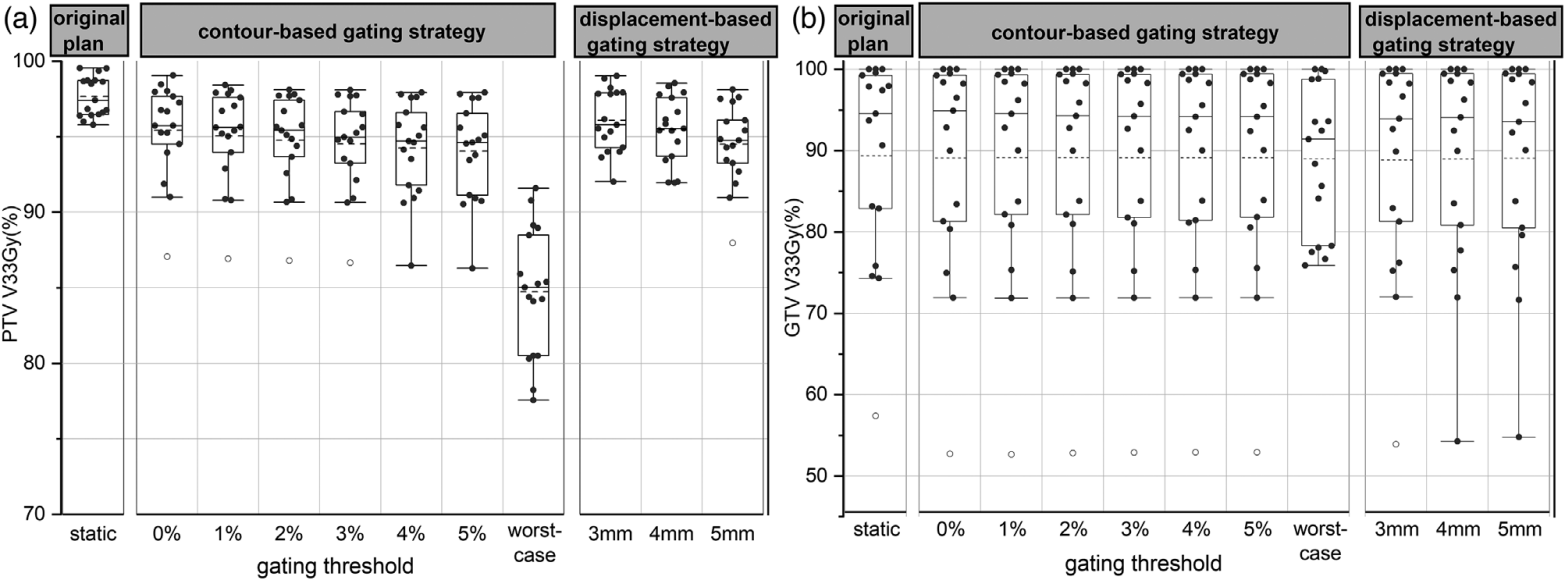
V33 Gy of the PTV (a) and GTV (b) of the VMAT plans at different gating thresholds in the tumor contour‐based and tumor displacement‐based gating strategies. The top and bottom of the box indicate the 25th and 75th percentile and the circle indicates the outlier. The mean and medium value are indicated by dotted line and solid line, respectively.

The PTV V33Gy and GTV V33Gy of the original and motion VMAT plans for 17 patients when applying different gating thresholds are shown in Figure 5. Table 5 presents the statistical comparisons between the original plan and motion plans. The results demonstrated a statistically significant decrease in PTV coverage and spinal cord Dmax for motion plans when compared to the original plan. There were no significant differences in the other parameters. For the worst‐case scenario, there were significant differences for PTV V33Gy, duodenum Dmax, duodenum V12.5Gy, and duodenum V18Gy.

**TABLE 5:** Dosimetry comparisons for VMAT plans.

			Tumor contour‐based gating strategy							Tumor displacement‐based gating strategy		
		Original plan	0%	1%	2%	3%	4%	5%	worst	3 mm	4 mm	5 mm
		Mean±SD	Mean±SD	Mean±SD	Mean±SD	Mean±SD	Mean±SD	Mean±SD	Mean±SD	Mean±SD	Mean±SD	Mean±SD
	Parameter	(Median)	(Median)	(Median)	(Median)	(Median)	(Median)	(Median)	(Median)	(Median)	(Median)	(Median)
GTV	V33Gy (%)	89.4±12.6	89.1±13.3	89.2±13.2	89.1±13.2	89.1±13.2	89.1±13.2	89.1±13.2	89.0±9.1	88.8±13.3	89.0±13.1	89.0±13.0
		(94.6)	(94.9)	(94.5)	(94.3)	(94.2)	(94.2)	(94.2)	(91.4)	(93.9)	(94.1)	(93.5)
	*P* value_corr_		1.000	1.000	1.000	1.000	1.000	1.000	1.000	0.676	1.000	1.000
PTV	V33Gy (%)	97.7±1.3	95.4±3.1	95.1±3.1	94.8±3.1	94.5±3.1	94.3±3.2	94.1±3.2	84.7±4.2	96.1±2.1	95.5±2.2	94.5±2.7
		(97.4)	(95.7)	(95.6)	(95.4)	(95.0)	(94.7)	(94.6)	(85.0)	(95.8)	(95.5)	(94.8)
	*P* value_corr_		**<0.001**	**<0.001**	**<0.001**	**<0.001**	**<0.001**	**<0.001**	**<0.001**	**<0.001**	**<0.001**	**<0.001**
Duodenum	Dmax (cGy)	3096±110	3103±144	3105±149	3105±149	3105±148	3104±146	3106±148	3475±214	3104±129	3106±131	3107±131
		(3137)	(3150)	(3139)	(3125)	(3125)	(3125)	(3125)	(3540)	(3133)	(3122)	(3122)
	*P* value_corr_		1.000	1.000	1.000	1.000	1.000	1.000	**0.002**	1.000	1.000	1.000
	V12.5Gy (cc)	8.9±0.7	9.3±1.4	9.4±1.6	9.5±1.7	9.5±1.7	9.5±1.7	9.5±1.7	12.7±4.6	9.3±1.1	9.4±1.3	9.5±1.5
		(9.1)	(9.2)	(9.4)	(9.4)	(9.4)	(9.4)	(9.4)	(11.1)	(9.3)	(9.5)	(9.5)
	*P* value_corr_		1.000	0.984	1.000	0.654	0.567	0.620	**0.002**	0.348	0.194	0.299
	V18Gy (cc)	3.0±0.7	3.2±0.9	3.3±0.9	3.3±0.9	3.3±0.9	3.3±0.9	3.4±1.0	6.2±3.5	3.2±0.7	3.3±0.8	3.4±0.9
		(3.2)	(3.1)	(3.2)	(3.2)	(3.3)	(3.3)	(3.3)	(5.1)	(3.2)	(3.2)	(3.3)
	*P* value_corr_		1.000	1.000	1.000	0.909	0.694	0.619	**0.004**	0.195	0.170	0.195
Stomach	Dmax (cGy)	2639±653	2622±648	2627±654	2627±658	2626±661	2621±663	2624±664	2666±907	2640±657	2634±662	2630±667
		(2828)	(2829)	(2832)	(2832)	(2835)	(2836)	(2839)	(2989)	(2831)	(2835)	(2840)
	*P* value_corr_		1.000	1.000	1.000	1.000	1.000	1.000	1.000	1.000	1.000	1.000
	V18Gy (cc)	4.7±3.5	4.6±3.5	4.6±3.6	4.6±3.5	4.6±3.5	4.6±3.6	4.6±3.6	6.5±6.3	4.7±3.6	4.7±3.6	4.7±3.6
		(3.8)	(3.6)	(3.6)	(3.7)	(3.7)	(3.7)	(3.7)	(4.2)	(3.7)	(3.8)	(3.9)
	*P* value_corr_		1.000	1.000	1.000	1.000	1.000	1.000	0.290	1.000	1.000	1.000
Liver	V21Gy (cc)	1548±502	1548±502	1548±502	1548±502	1549±502	1549±502	1549±502	1550±502	1549±502	1549±502	1549±502
		(1458)	(1458)	(1458)	(1458)	(1458)	(1458)	(1458)	(1458)	(1458)	(1458)	(1458)
	*P* value_corr_		1.000	1.000	1.000	1.000	1.000	0.625	0.625	1.000	1.000	0.625
Small bowel	Dmax (cGy)	2463±670	2445±673	2444±675	2444±678	2445±680	2447±682	2449±683	2680±751	2448±676	2449±680	2448±682
		(2921)	(2921)	(2923)	(2928)	(2931)	(2933)	(2934)	(2827)	(2916)	(2920)	(2926)
	*P* value_corr_		0.809	1.000	1.000	1.000	1.000	1.000	0.056	0.958	1.000	1.000
	V25Gy (cc)	0.5±0.5	0.4±0.5	0.4±0.5	0.5±0.5	0.5±0.5	0.5±0.5	0.5±0.5	1.6±2.2	0.4±0.5	0.5±0.5	0.4±0.5
		(0.2)	(0.1)	(0.1)	(0.2)	(0.2)	(0.2)	(0.2)	(0.3)	(0.2)	(0.2)	(0.2)
	*P* value_corr_		1.000	1.000	1.000	1.000	1.000	1.000	0.210	1.000	1.000	1.000
Left kidney	Dmean (cGy)	391±176	388±173	389±173	389±173	389±173	389±173	389±173	410±184	388±173	389±173	389±173
		(362)	(362)	(363)	(364)	(364)	(364)	(364)	(382)	(363)	(363)	(364)
	*P* value_corr_		1.000	1.000	1.000	1.000	1.000	1.000	1.000	1.000	1.000	1.000
Right kidney	Dmean (cGy)	419±194	417±192	418±193	418±193	418±193	418±193	418±193	453±192	418±192	418±192	419±193
		(381)	(377)	(379)	(380)	(381)	(381)	(382)	(399)	(382)	(383)	(384)
	*P* value_corr_		1.000	1.000	1.000	1.000	1.000	1.000	0.067	1.000	1.000	1.000
Spinal Cord	Dmax (cGy)	942±351	936±349	936±349	935±349	935±348	934±348	934±348	966±363	935±349	934±348	934±348
		(878)	(874)	(874)	(874)	(874)	(873)	(873)	(877)	(874)	(874)	(873)
	*P* value_corr_		**0.002**	**0.002**	**0.002**	**0.002**	**0.004**	**0.009**	0.115	**<0.001**	**0.002**	**0.005**

*Note*: *P* value_corr_ is the *P* value after Bonferroni correction. The *P* values of <0.05 were presented in bold.

Table 6 summarizes the *P* values of the PTV V33Gy comparisons between any two gating thresholds in the tumor contour‐based gating strategy. As shown, when the gating threshold was greater than 2%, the PTV V33Gy was significantly lower than that at the 0% gating threshold. No significant difference was found for PTV coverage between any two gating thresholds within 3–5%. The worst‐case scenario plan was significantly different from all the motion plans except for the 5% gating threshold. In the tumor displacement‐based gating strategy, the 4 mm gating threshold resulted in a PTV coverage significantly better than the 5 mm threshold (*P* = 0.011) but worse than the 3 mm threshold (*P* = 0.011).

**TABLE 6:** PTV V33Gy comparisons between motion VMAT plans at different gating thresholds in the tumor contour‐based gating strategy.

Gating threshold	1%	2%	3%	4%	5%	Worst‐case
0%	1.000	0.674	**0.024**	**<0.001**	**<0.001**	**<0.001**
1%		1.000	0.991	**0.047**	**0.001**	**<0.001**
2%			1.000	0.609	**0.021**	**<0.001**
3%				1.000	0.609	**0.001**
4%					1.000	**0.036**
5%						0.901

*Note*: The *P* values of <0.05 were presented in bold.

**FIGURE 6:**
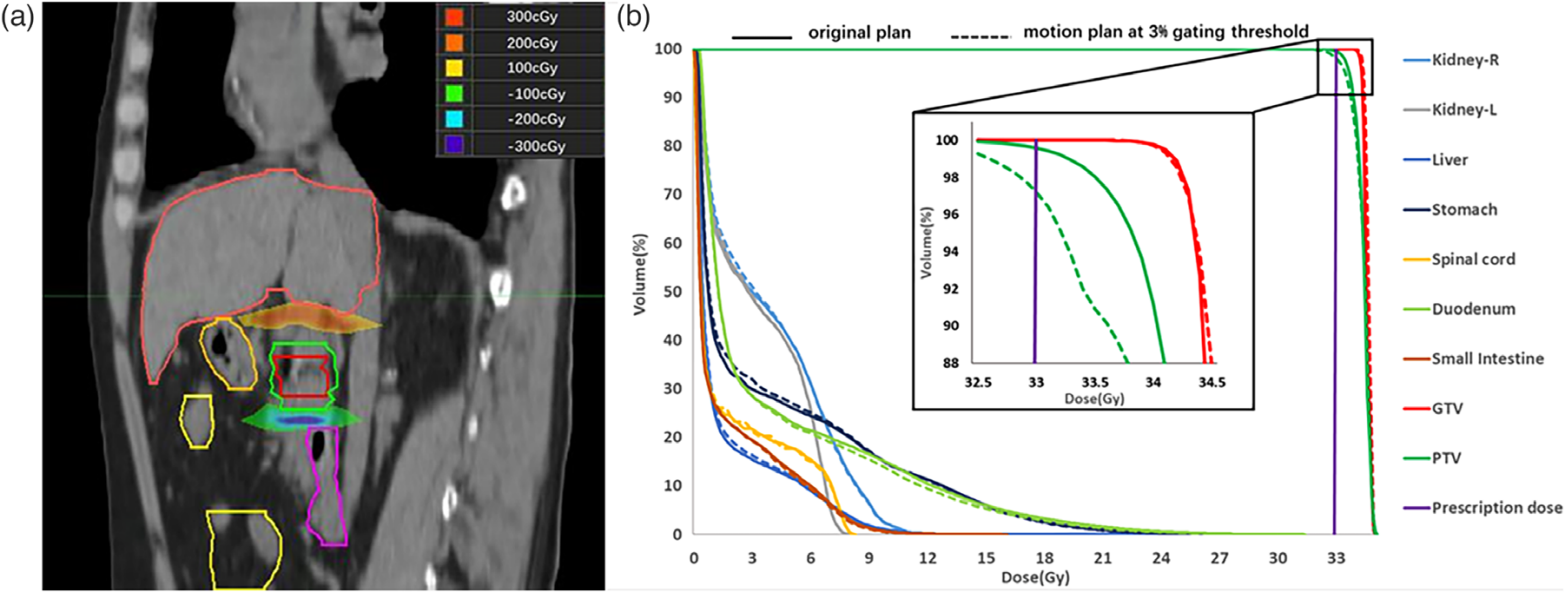
The change in dose distribution for a VMAT motion plan at the 3% gating threshold. (a) The dose difference between the motion plan and the original plan; (b) the DVHs of the original plan (solid line) and the motion plan (dashed line).

As an example, Figure 6a shows the dose difference between the motion plan at the 3% gating threshold and the original plan. As observed, the major difference occurred in the superior and inferior boundaries of GTV and PTV, resulting in underdose at the inferior boundary and overdose at the superior boundary in the motion plan. Figure 6b shows the DVH of the original plan and the motion plan. The difference was obvious for the PTV but not for the GTV.

### 3.4 Treatment efficiency

**FIGURE 7:**
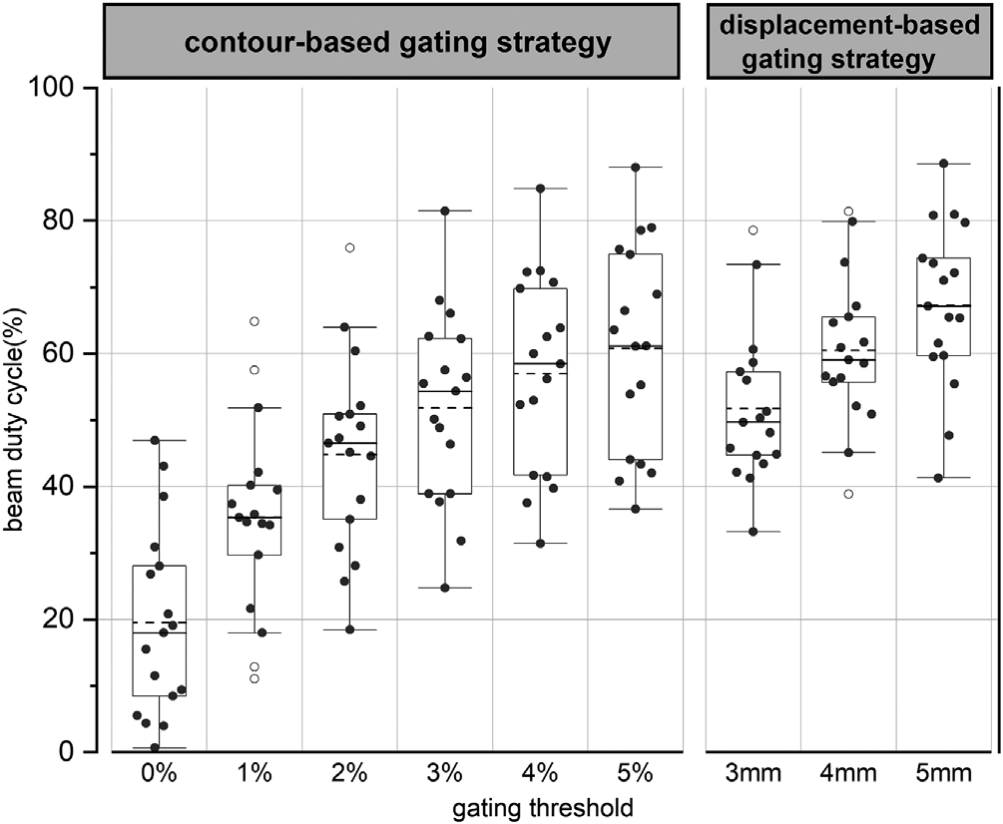
The beam duty cycle at different gating thresholds. The top and bottom of the box indicate the 25th and 75th percentile and the circle indicates the outlier. The mean and medium value are indicated by dotted line and solid line, respectively.

The beam duty cycles calculated for the motion plans are shown in Figure 7. The beam duty cycles were 19.5±14.3% (18.0%), 35.4±14.5% (35.3%), 44.85±14.58% (46.5%), 51.8±14.5% (54.3%), 57.0±14.9% (58.4%), and 60.8±15.6% (61.1%) at gating thresholds of 0, 1, 2, 3, 4, and 5% in the tumor contour‐based gating strategy and 51.7±11.5% (49.7%), 60.5±11.2% (59.1%), and 67.3±12.4% (67.1%) at gating thresholds of 3, 4, and 5 mm in the tumor displacement‐based gating strategy. Table 7 summarizes the beam duty cycle comparisons between motion plans at different gating thresholds in the tumor contour‐based gating strategy. As shown, the beam duty cycles at a gating threshold of 0–2% were significantly lower than gating thresholds of 4% and 5%, while no significant difference was found between the 3% and 4% gating thresholds. In the tumor displacement‐based gating strategy, the 4 mm gating threshold resulted in a duty cycle significantly better than the 3 mm threshold (*P* = 0.011) but worse than the 5 mm threshold (*P* = 0.011).

**TABLE 7:** Duty cycle comparisons between different gating thresholds in the tumor contour‐based gating strategy.

Gating threshold	1%	2%	3%	4%	5%
0%	1.000	**0.027**	**<0.001**	**<0.001**	**<0.001**
1%		1.000	**0.027**	**<0.001**	**<0.001**
2%			1.000	**0.027**	**<0.001**
3%				1.000	**0.027**
4%					1.000

*Note*: The *P* values of <0.05 were presented in bold.

## 4 DISCUSSION

In cine MRI‐gated radiation therapy, a greater gating threshold relaxes the beam‐on condition and can shorten the treatment time but at the cost of reduced treatment accuracy. In contrast, lowering the gating threshold would improve treatment accuracy but prolong the treatment time. Therefore, it is important to balance effectiveness and efficiency. In this study, the tumor displacements are acquired by the NCC‐based template matching algorithm to independently validate the clinically used tumor contour‐based gating strategy. Instead of investigating motion phantoms,^36^ this study is directly based on clinical data and concludes that a threshold up to 5% may be acceptable in terms of GTV coverage in breath‐hold pancreatic cancer radiotherapy. Considering the treatment efficiency, the gating threshold might be no less than 3%. Moreover, a displacement‐based gating strategy was proposed and may serve as a potential alternative to the tumor contour‐based gating strategy. In the tumor displacement‐based gating strategy, a 4 mm gating threshold might be a good choice that can reasonably balance the dose delivery accuracy and efficiency.

In the tumor contour‐based gating strategy, as currently implemented in the clinic, the target was real‐time auto‐contoured by deforming the contour in the reference frame to the current motion frame. Some specific tracking algorithms have been developed for particular applications to account for the different deformation patterns of targets and OARs.^37^, ^38^ In this study, we used the NCC‐based template matching algorithm to independently acquire the target displacement. The validation study showed that the displacement calculation error, for example, 0.62 ± 0.25 (0.55) mm in the SI direction, was comparable to the magnitude of the inter‐observer variability in the centroid displacement and was relatively small when compared to the spatial resolution of the cine MR images, so the acquired displacements were directly used to generate motion plans.

In this study, the plans were generated in a Pinnacle TPS. Regarding the planning technique itself, several studies have indicated that plans created in the MR‐Linac TPS were dosimetrically comparable to those traditional Linac‐based plans.^39^, ^40^, ^41^ The aim of this study is to evaluate the motion impact on the dose distribution, so we did not focus on plan deliverability. To further consider the impact of any specific MR‐Linac system, one must model the interplay of gantry, MLC, and respiratory motion during radiation delivery. The interplay, however, cannot be accounted for even if the plan is generated in the MRI‐Linac TPS because of the lack of real‐time 3D tumor motion data and real‐time recording of gantry and MLC locations. Therefore, the dose accumulation in the motion plan was achieved by a simplistic rigid dose shift. In conclusion, which TPS was used should not make a big difference in the current study outcome. Step‐and‐shoot IMRT is the existing treatment approach on the MR‐Linac system. Considering that VMAT has been increasingly used, we also evaluated VMAT plans. The difference in the dosimetric outcome between the MR‐Linac IMRT and traditional‐Linac VMAT plans has been investigated by Redler et al.^40^ In that study, eight SBRT patients who were previously treated with VMAT on TrueBeam were replanned using fixed‐field IMRT on MR‐Linac. The authors concluded that the dosimetric outcome was consistent between the two types of plans. In this study, the IMRT and VMAT planning techniques have been investigated. Other techniques, such as partial‐arc,^42^, ^43^, ^44^ could also be implemented to improve the treatment plan quality. Considering the limited study scope, we could not go through all possible planning techniques. But it should not change the study outcome about the gating effects.

The 2D sagittal cine MRI offers advantages in soft tissue contrast while ensuring the imaging speed to meet the clinical requirements. However, it suffers from complications arising from out‐of‐plane motion in the left‐to‐right direction, which can result in inconsistency between the detected target shift and real 3D motion. Keiper et al. ^45^ noticed that the tracking accuracy depended on the acquisition plane and the motion pattern and concluded that choosing an appropriate tracking region is very important. In the current study, we set the tracking region in the middle plane of the tumor, which hopefully can mitigate the target tracking variation caused by the out‐of‐plane effect. In the future, once real‐time 3D MR imaging becomes available in radiation oncology practice, investigations about the extent of the out‐of‐plane motion and how it affects the target tracking accuracy should be conducted. The method of acquiring several parallel slices ^23^, ^46^ or interleaved slices ^47^, ^48^, ^49^ have been investigated to monitor real‐time 3D motion. The feasibility of using the 2D orthogonal cine‐MR images to monitor the 3D tumor motion has been validated on a digital 4D lung phantom.^50^ In clinic, the technique of using orthogonal images to monitor the tumor 3D motion ^51^ has been implemented in Elekta's Unity,^52^ which is another MR‐Linac. The idea of using two orthogonal images will largely address the out‐of‐plane motion effect by providing the tumor 3D displacement.

A major limitation of the current study is that the motion plan was acquired by adding up isocenter‐shift plans with various isocenter‐displacement values, which were calculated assuming a rigid anatomic motion. In reality, however, the moving parts are mainly the tumor and nearby OARs, while the bony anatomy remains stationary, which explains why there was a significant difference in spinal cord Dmax between the original plan and motion plans. Indeed, it is usually more reliable and accurate to accumulate the dose by deformable registration rather than rigid registration.^53^ However, deformation registration requires real‐time 3D motion anatomy acquired during radiation delivery, which was not available in the current study. Once real‐time 3D MRI becomes available in the future, a more rigorous investigation can be conducted. In addition, cine MRI data were acquired from an MR‐guided cobalt radiation therapy system, and the breathing amplitude and frequency may change due to different treatment durations. However, since the critical information used in this study was the time proportions corresponding to different target displacements instead of the absolute time lengths, it should not make a large difference in the study outcome.

## 5 CONCLUSION

In the tumor contour‐based gating strategy, the dose delivery accuracy deteriorates while the dose delivery efficiency improves with increasing gating thresholds. To ensure treatment efficiency, the gating threshold might be no less than 3%. A threshold up to 5% may be acceptable in terms of the GTV coverage. The displacement‐based gating strategy may serve as a potential alternative to the tumor contour‐based gating strategy, in which the gating threshold of approximately 4 mm might be a good choice for reasonably balancing the dose delivery accuracy and efficiency.

## AUTHOR CONTRIBUTIONS

Yuyan Dong: Study design, data analysis and manuscript drafting; Panpan Hu: Study design, data analysis and manuscript revision; Xiaoyang Li: Data analysis and manuscript revision; Wei Liu: Data analysis and manuscript revision; Bing Yan: Data analysis and manuscript revision; Fei Yang: Data collection, statistical analysis and manuscript revision; John Chetley Ford: Data collection and manuscript revision; Lorraine Portelance: Data collection and manuscript revision; Yidong Yang: Study guidance, manuscript revision and financial support.

## CONFLICT OF INTEREST STATEMENT

The authors have nothing to disclose.

## ETHICS STATEMENT

This study followed the protocol approved by the University of Miami (IRB # 20160817).
